# Retraction Note: Pharmacokinetics, Biodistribution, and Anti-Angiogenesis Efficacy of Diamino Propane Tetraiodothyroacetic Acid-conjugated Biodegradable Polymeric Nanoparticle

**DOI:** 10.1038/s41598-023-43642-5

**Published:** 2023-10-03

**Authors:** Weikun Li, Murat Yalcin, Dhruba J. Bharali, Qishan Lin, Kavitha Godugu, Kazutoshi Fujioka, Kelly A. Keating, Shaker A. Mousa

**Affiliations:** 1 The Pharmaceutical Research Institute, Albany College of Pharmacy and Health Sciences, Rensselaer, NY USA; 2 Department of Physiology, Veterinary Medicine Faculty, Uludag University, Bursa, Turkey; 3 Center for Functional Genomics, University at Albany SUNY, Albany, NY USA

Retraction of:* Scientific Reports* 10.1038/s41598-019-44979-6, published online 21 June 2019

The Editors have retracted this article.

An investigation by Albany College of Pharmacy and Health Sciences has concluded that the content of four images in Fig. 10b appears to have been misrepresented.

Specifically:

- The PBS control appears identical to b-FGF + 7 in Fig. 4^1^ and to PBS image in Fig. 4 of^2^.
- The b-FGF image appears identical to VEGF in ‘corrected’ Fig. 2A of^3^.
- The b-FGF + DAT image appears identical to b-FGF + 5 in Fig. 4 of^1^ and to b-FGF + Au-glucose in Fig. 4 of^2^.
- The b-FGF + N-DAT image appears identical to b-FGF + tetrac in Fig. 4 of^1^ and to b-FGF + Ag-DAPHP in Fig. 4 of^2^.

The Editors therefore no longer have confidence in the results and conclusions presented.

Murat Yalcin, Qishan Lin, Kavitha Godugu, Kazutoshi Fujioka, Kelly A. Keating & Shaker A. Mousa did not respond to the correspondence from the Editors about this retraction. The Editors were not able to confirm the current contact details for Weikun Li and Dhruba J. Bharali.
